# Maotai Ameliorates Diethylnitrosamine-Initiated Hepatocellular Carcinoma Formation in Mice

**DOI:** 10.1371/journal.pone.0093599

**Published:** 2014-04-01

**Authors:** Xu Yi, Li Long, Chunzhang Yang, Yingying Lu, Mingliang Cheng

**Affiliations:** 1 Guiyang Medical College Hospital, GuiYang, Guizhou, China; 2 Surgical Neurology Branch, National Institute of Neurological Disorders and Stroke, National Institutes of Health, Bethesda, Maryland, United States of America; 3 Center for Therapeutic Research of Hepatocellular Carcinoma, Beijing 302 Hospital, Beijing, China; Institut für Pathologie, Greifswald, Germany, Germany

## Abstract

Consumption of alcohol is closely related to liver disease, such as hepatic fibrosis or even hepatocellular carcinoma (HCC). However, epidemiological and experimental studies indicated that consumption of Maotai, one of the famous liquors in China, exhibits no significant correlation with hepatic fibrosis or cirrhosis as other beverage sources do. This study detected the relationship of Maotai consumption and HCC development in a diethylnitrosamine (DEN)-initiated HCC animal model. DEN was given to mice at a dose of 100 mg/kg, ip, and 50 mg/kg, ip in the following week. Mice were simultaneously given Maotai or an equal amount of ethanol (53%, 5 ml/kg/day, 5days/week for up to 35weeks). At 3-week and 35- week of the experiment, serum and livers were collected for biochemical and histopathological examination of liver injury and incidence of HCC. Real-time RT-PCR, immunohistochemistry and Western blotting were used to examine the expression of metallothionein-1/2 (MT-1/2), NF-E2-related factor 2 (Nrf2), glutamate-cysteine ligase catalytic subunit (GCLC) and modified subunit (GCLM). We identified tissue damage and dysfunction of liver in ethanol + DEN-treated mice, whereas the extent of injury was reduced in Maotai+ DEN –treated mice. Significant Glypican-3(GPC3) expression and precancerous injury or HCC were seen in approximately 50% of mice with ethanol+ DEN, but barely be seen in Maotai + DEN-treated mice. A higher expression of MT-1/2, Nrf2 and GCLC could be seen in Maotai + DEN-treated mice. Thus, Maotai liquor ameliorates the formation of DEN-induced HCC in mice, and the protection mechanism is possibly related with the activation of anti-oxidation factors, such as MTs, Nrf2 and GCLC.

## Introduction

Hepatocellular carcinoma (HCC) is the most common primary malignancy of the liver with increasing incidence over the last several decades, and third cancer-related mortality worldwide [1], [2]. Hepatocarcinogenesis is considered to be a multi-gene and multi-step disease process and likely involves various pathogenic factors, such as hepatitis B or hepatitis C infection, aflatoxin, and chronic, heavy ethanol consumption [1], [3].It is well known that chronic alcohol consumption has long been associated with progressive liver disease toward the development of hepatic cirrhosis and the progression to HCC, especially in developed countries [3]–[5].Prior studies indicated that alcohol synergizes with HBV, HCV and carcinogenic agents, increases the occurrence of liver cirrhosis and HCC[6]–[10]. Chronic ethanol feeding can accelerate HCC progression in male mice than females in the setting of being initiated by DEN [10]. However, light alcoholic beverage consumption shows beneficial effects to reduce cardiovascular diseases, risk of diabetes, and may have protective effects toward cognitive function in women, non-alcoholic fatty liver disease, carcinogenesis and osteoporosis [11]–[14]. Maotai is a traditional Chinese distilled liquor, containing 53% alcohol (v/v), enjoyed by many people all over the world [15].An epidemiological study was performed on workers consuming Maotai longer than ten years during the course of production to taste its quality, the results showed there was no evidence of hepatic fibrosis or cirrhosis in the special crowd, as compared to other beverage sources [15]. Further experimental studies in animals with short-term oral administration of Maotai or Hep3B cells with exposure of Maotai residue without ethanol showed that Maotai induced remarkable higher metallothioneins and glutathione levels in the liver and produced less liver pathology compared with equal amount of ethanol administration [11], [15]–[18]. All these results suggest that Maotai could be different from ordinary ethanol and could be less toxic to the liver than the equal alcohol contents from other beverage sources.

The effects and the possible mechanism of long and constant drinking of Maotai on the hepatocarcinogenesis remain unclear. It is well known that oxidative stress plays a key role in alcohol-related hepatic injury, and promotes the development and progression of hepatic fibrosis or cirrhosis, an important process related to hepatocarcinogenesis [19].In present study, we identified specific induction of antioxidative stress-related genes through Maotai consumption, suggesting that Maotai ameliorates cancer formation through affecting oxidative stress pathways. In this paper, special attention was paid to metallothioneins (MTs), the intracellular small, cysteine-rich and metal-binding proteins. MTs could be induced at high levels by oxidative stress and participate in an array of protective responses [20]–[22]. MTs coded by 4 different genes in mammals are highly homologous and evolutionarily conserved [21]. MT-1 and MT-2 are ubiquitously expressed and are present almost in all types of soft tissues [22]–[24]. MTs play an important role in protection against alcoholic liver injury through inhibition of oxidative stress [20], [21], [24]. Silencing of MTs in both rodent and human HCC suggests their potential role in predisposing hepatocytes to neoplastic transformation especially after toxic insults [23]. Except for MTs, Nuclear factor erythroid 2-related factor 2 (Nrf2) is a master cellular transcription factor that has a significant role in cellular response to oxidative stress, and serves to maintain intracellular redox homeostasis. The Nrf2-Keap1 antioxidant signaling pathway is important in protecting liver from various stimuli [25]–[27]. The role of Nrf2 in protecting liver damage has been extensively documented using genetic Nrf2 modulation animal models, as well as Nrf2 activation via pharmacological activators [28], [29]. Glutamate-cysteine ligase (GCL) is a key regulatory enzyme in the synthesis of glutathione, an important endogenous antioxidant. GCLC and GCLM respectively are catalytic and modified subunits of GCL and also are important Nrf2-ARE-targeted genes [23], [30], [31].This study utilized male C57BL/6J mice initiated by DEN to investigate and show for the first time that the effects of long-term drinking of Maotai on hepatocarcinogenesis. The results indicated that Maotai ameliorates the formation of HCC in mice. Real-time RT-PCR, immunohistochemistry and Western blotting were used to examine the expression of MT-1/2, Nrf2 and Nrf2-targeted genes GCLC and GCLM. The results clearly revealed the markedly increased MTs, Nrf2 and GCLC in Maotai+ DEN-treated mice.

## Materials and Methods

### Animals and ethical approval

Male C57BL/6J mice, 5–8 weeks old weighing 20–23 g were obtained from the Third Military Medical University(Chongqi, China).Institutional and national guidelines for the care and use of animals were followed and all experimental procedures involving animals were approved by the IAEC (institutional animal ethical committee) of Gui Yang Medical College (Permit Number: SYXK2012-0001). ALL animals were housed in the facilities with individual ventilated caging system in a 12-hr light-dark cycle. Animals were allowed free access to sterilized tap water. Mice were closely monitored by the body weights and general health. All operation was performed under sodium pentobarbital anesthesia, and all efforts were made to minimize suffering.

### Reagents and animal experiments

Maotai (53% ethanol, v/v) was obtained from Maotai Company (Guizhou, China) and absolute ethanol was obtained from Sinopharm (Shanghai, China). DEN was obtained from Sigma (Aldrich). Mice were divided into six groups, included: Control group (n = 22), Maotai group (n = 20), Ethanol (n = 20), Maotai+ DEN group (n = 24), Ethanol+ DEN group (n = 26) and DEN group (n = 22). Maotai or 53% ethanol (v/v) in water was given orally at a dose of 5 ml/kg by gavage. Mice were monitored 5 days/week for up to 35weeks. The mice in DEN group were given DEN at doses of 100 mg/kg, ip, and 50 mg/kg, ip in the following week and normal breed in the future. In addition to the processing of DEN group, the mice in Maotai +DEN group and ethanol+ DEN group were respectively given Maotai liquor and ethanol at the same time, at doses of 5 ml/kg by intragastric incubation 5 days/week for up to 35 weeks. Untreated animals were used as controls.

Serum and liver tissue samples were collected at the end of 3-week and 35-week treatment. Liver/body weight (BW) ratio was calculated according to the formula [32]:Liver/body weight(BW) ratio  = [mice liver weight(g)/mice body weight (g)]×100%.

### Histopathology

All liver tissue samples were embedded in paraffin after being fixed in 10% formalin for 24–48 h, and then 4-μm slices were made. We then detected the liver histological changes, such as the lesions of hepatocytes, fibrogenesis and the evaluation of liver structural change by using hematoxylin and eosin (H&E), Masson and Reticular fiber stain [33].

### Measure of level of ALT/AST in serum and MDA in liver homogenate

Detection of alanine transaminase (ALT) and aspartate transaminase (AST) in serum were performed by an fully automatic biochemical analyser (Siemens Advia 1650). Malondialdehyde (MDA) in liver homogenate was tested by the Enzyme linked immunosorbent assay (Becton, Dickinson) according to manufactor' s protocol.

### Immunohistochemistry

Immunohistochemistry studies were performed using EnVision System DAKO according to the manufactor's protocol. Tissue sections were de-paraffinized, rehydrated, and proceed for antigen retrieval using heat treatment in the presence of Tris-EDTA solution (DAKO), histological sections were then placed in 3% H_2_O_2_ for 20minute to eliminate endogenous peroxidase activity. The sections were probed with primary antibodies through the night at 4°C and the signal was developed with EnVision detection kit from DAKO. Immunostaining labeling intensities were defined as the number and the staining degree of positive cells:+less than 10% of the cells were positive and positive weakly; ++10%–50% of the cells were positive and positive medium, +++50–80% of the cells were positive and strong positive, ++++more than 80% cells were positive[34].The primary antibodies used in this study include anti-glypican-3(1∶500,ABBIOTEC),anti-MT(1∶50,DAKO), anti-Nrf2(1∶100,Abcam)

### Real-time RT-PCR Analysis

Total RNA was isolated from liver samples with Trizol regent (Tiangen), followed by purification and was reverse transcribed with MuLV reverse transcriptase (Thermo, Lithuania) and oligo-dT primers. The forward and reverse primer sequences for selected genes were designed with Primer 3 online software and are listed in Table 1. Gene expression was then determined using 2×SYBR green PCR master mix (Biomiga). The relative differences in expression between groups were calculated using the ΔΔCt method.

**Table 1:** Primer sequences for real-time RT-PCR analysis.

Genes	Number	Gene Bank	
		Forward	Reverse
β-actin	NM_007393.3	GGCCAACCGTGAAAAGATGA	CAGCCTGGATGGCTACGTACA
MT1	NM_013602.3	AATGTGCCCAGGGCTGTGT	GCTGGGTTGGTCCGATACTATT
MT2	NM_008630.2	TGTGCCTCCGATGGATCCT	GCAGCCCTGGGAGCACTT
Nrf2	NM_010902.3	CGAGATATACGCAGGAGAGGTAAGA	GCTCGACAATGTTCTCCAGCTT
GCLC	NM_010295.2	CCTCCTCCTCCAAACTCAGATA	CCACAAATACCACATAGGCAGA
GCLM	NM_008129.4	CACAATGACCCGAAAGAACTG	AGACTTGATGATTCCCCTGCT

### Western blot

Total protein was extracted and quantified using the Bradford protein quantification kits (Dingguo Changsheng). Eighty microgram of total protein was resolved by sodium dodecyl sulfate-polyacrylamide gel electrophoresis (SDS-PAGE).The protein was transferred onto PVDF blotting membranes (Millipore) and were incubated with primary antibody overnight at 4°C. On the second day, the signal was developed with electrochemiluminescence detection kit (Millipore) after incubation with appropriate secondary (Dingguo Changsheng).Primary antibodies used in this study include MT (1∶200, Santa Cruz), Nrf2 (1∶800, Abcam), GCLC (1∶500, Abcam) and GCLM (1∶4000, Abcam), and β-actin (Cell Signaling).

### Statistical analyses

Data analysis was performed using SPSS 19.0 software. Data are expressed as the mean± SD. For comparisons of gene or protein expression between the multiple groups, the one-way analysis of variance was performed. Radit analysis was performed for the analysis of ordinal data, *P*<0.05 was credited with significant differences.

## Results

### Effect of Maotai liquor on body weight, Liver weight and Anatomy

**Figure 1:**
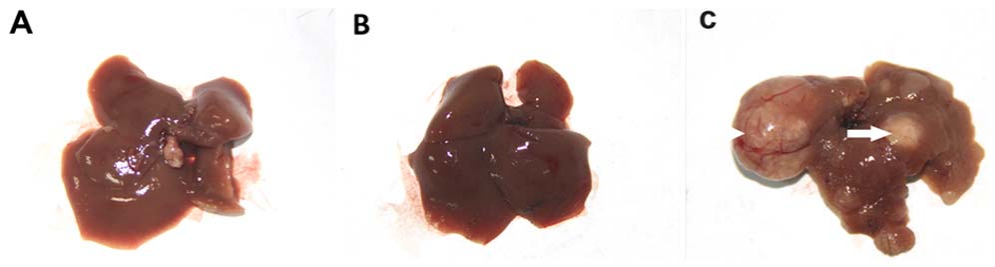
Representative images of the mice liver specimen at 35-week. A: Control group; B:Maotai+ DEN group; C: Ethanol+ DEN group. No obvious macroscopic hepatic pathological changes was seen in control group and Maotai+ DEN group. The liver in ethanol+ DEN treated group almost showed swelling, cirrhosis, brown and produced unequal sized gray nodules(arrows indicate the tumor node).

At the end of the experiments, two mice died in control group, four mice died in Maotai + DEN group, six mice died in ethanol+ DEN group, and two mice died in DEN group. Maotai or ethanol treatment had no significant effect on the growth of mice. The body weight of mice in the group treated by Single DEN (22.3±1.06 g vs 29.4±3.3 g) or Maotai+ DEN (22.4±1.07 g vs 27.3±1.29 g) was similar with the mice in control group. However, the ethanol + DEN treatment resulted in obvious loss of body weight. The final weight (19.9±2 g) was lower than the initial weight (22.5±1.06 g). Statistical analysis showed that the body weight of ethanol + DEN group mice was significantly lower than that each of other groups (*p*<0.001, Table 2). Except ethanol + DEN group, other treatment groups have the same liver appearance, no obvious macroscopic hepatic pathological changes. The liver in ethanol+ DEN treated group showed swelling, cirrhosis, brown and produced unequal sized gray nodules (the maximum diameter could reach 0.5 to 1.0 cm, Figure 1). The liver/body weight(BW)ratio in ethanol+ DEN group increased significantly than other groups(*P*<0.001, Table 2).

**Table 2:** Animal/Liver Weights and Liver/BodyWeight(BW)Ratio(final) at Necropsy.

Group	Number	Initial body weight(g)	Final body weight(g)	Liver(g)	Liver/BW ratio
Control	10	23.0±1.67	28.6±1.18*	1.36±0.22*	4.76±0.73*
Maotai	10	22.1±1.05	29.3±2.87*	1.34±0.22*	4.55±0.33*
Ethanol	10	22.6±1.09	30.8±2.13*	1.40±0.22*	4.53±0.58*
Maotai+ DEN	10	22.4±1.07	27.3±1.29*	1.33±0.21*	4.86±0.62*
Ethanol+ DEN	10	22.5±1.06	19.9±2	1.82±0.44	9.07±1.83
DEN	10	22.3±1.06	29.4±3.3*	1.46±0.36*	4.89±0.82*

Liver/BW ratio  = [mice liver weight(g)/mice body weight (g)]×100%;

*Significantly different from ethanol+ DEN group, *P*<0.05.

### Effect of Maotai liquor on histopathology of liver

**Figure 2:**
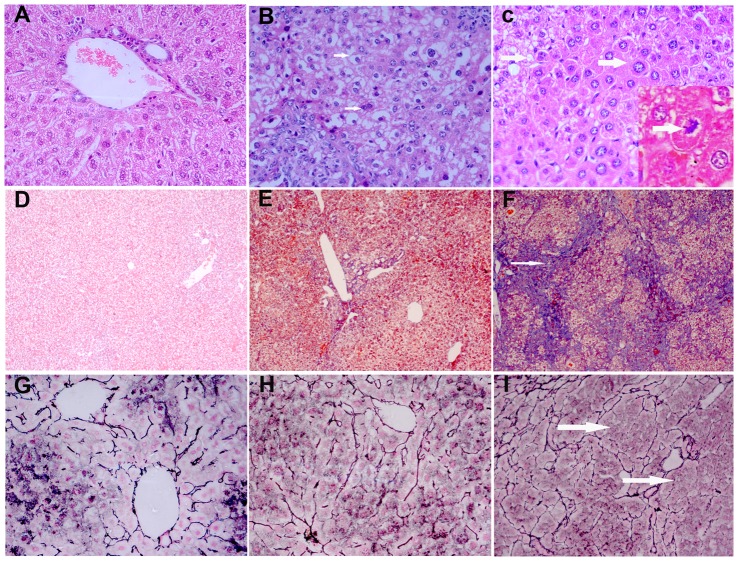
Pathological changes of liver in 3 groups mice at 35-week. A–C: Light microscopy of H&E staining in control group, Maotai+ DEN group and ethanol+ DEN group (400× magnification). Hepatocyte ballooning degeneration, edema and regeneration could be seen in Maotai+ DEN group, The lobular structure of hepatocytes in ethanol+ DEN group was destroyed with fatty degeneration, bridging necrosis, hepatocytes abnormal fission, atypical hyperplasia in ethanol+ DEN group; D–F: Light microscopy of Masson staining in control group, Maotai+ DEN group and ethanol+ DEN group (100×magnification).As the arrow indicate, significant liver fibrosis and pseudolobule formation in ethanol+ DEN group, but only moderate fibrosis formation in Maotai+ DEN group; G–I: Light microscopy of reticulin fiber staining in control group, Maotai+ DEN group and ethanol+ DEN group (400×magnification).Arrows indicate liver tissue structure obvious change and “floating trabeculae” in ethanol+ DEN group, but was normal in Maotai+ DEN group.

We then compared the liver histological changes by using H&E, Masson and Reticular fiber staining. Pathological identification for rodent tumors was according to the criteria published by the International Agency for Research on Cancer [35]. Hepatocytes in control group radiated out in around the central vein, and no fibroplasia could be seen. Fatty degeneration, bridging necrosis, hepatocytes abnormal fission, atypical hyperplasia or “floating trabeculae”,architecture often characteristic of HCC could be seen in the hepatic lobule in ethanol+ DEN group. In addition to these, a large number of inflammatory cells infiltration, significant fiber hyperplasia and the formation of pseudolobule existed in some liver tissues. The level of hepatic fibrosis in ethanol + DEN group was much more obvious compared to other groups (*p*<0.05) The lobular structure of Maotai group, ethanol group, Maotai+ DEN group and DEN group were still in integrity. Some hepatocyte ballooning degeneration, edema, regeneration, focal point necrosis and mild-to-moderate fibrosis could be seen in Maotai+ DEN group. (Figure 2, Table 3)

**Table 3:** The evolution of hepatic fibrosis in each group of mices

Group	n	Degree of hepatic fibrosis					Mean Ridit
		0	1	2	3	4	
Control	10	10	0	0	0	0	0*
Maotai	10	10	0	0	0	0	0*
Ethanol	10	5	5	0	0	0	0.5*
Maotai+ DEN	10	0	10	0	0	0	1*
Ethanol+ DEN	10	0	0	2	4	4	2.5
DEN	10	0	10	0	0	0	1*

*Significantly different from ethanol+ DEN group, *P*<0.05.

### Effect of Maotai liquor on expression level of glypican-3 (GPC3)

**Figure 3:**
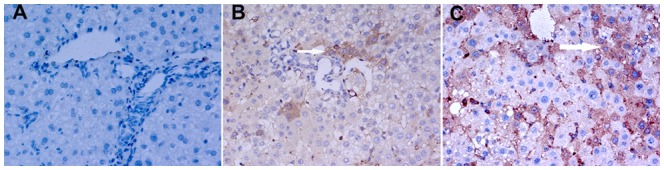
GPC3 expression of liver tissue in mice through Immunohistochemistry (400× magnification, allows indicate GPC3 expression cytolymph positive cells). A: The negative expression of GPC3 expression in control group mice liver; B: The small amount of positive expression of GPC3 was be seen in Maotai+ DEN group mice liver; C:The remarkable positive expression of GPC3 in ethanol+ DEN group mice liver.

GPC3 is highly expressed in HCC, while be less frequent in preneoplastic or entirely absent in normal liver tissue [36]–[38].It was reported that the frequency of GPC3 expression in AFP-negative HCC patients is as high as 90% [39].This has made GPC3 as an useful and specific tumor diagnostic marker in the diagnosis for HCC. In our study, Immunohistochemical assays showed that GPC3 was significantly expressed in ethanol+ DEN group, some liver tissues in Maotai+ DEN group and DEN group showed a lower expression and no any expression could be seen in control groups. (Figure 3)

### Effect of Maotai on the serum levels of ALT, AST and MDA in liver homogenates

ALT and AST serum values have been widely used as sensitive laboratory parameters of inclinical practice contributing to an understanding for liver injury[40].In our studies, ethanol group, DEN group, Maotai+ DEN group and ethanol+ DEN group separately showed increased ALT and AST levels than control group at 3-week (*P*<0.001, Table 4). Maotai group always remained similary ALT and AST level with control group both at 3-week and 35-week(*P* = 0.96)[15].At the 35-week, in addition to ethanol +DEN group, the ALT or AST levels in other groups were significantly decreased to normal level(*P*>0.05, Table 4), but ethanol+ DEN group still has a significantly higher ALT or AST levels than that in all other groups (*P*<0.05, Table 4).

**Table 4:** Serum ALT/AST and MDA in liver homogenate in each group in different periods(mean±SD).

Group	ALT (IU/L)	AST (IU/L)	MDA(nmol/mg)
3-week			
Control	44.5±1.6	102.3±6.9	7.57±0.2
Maotai	48.2±7.3	100.5±11.5	7.51±0.2
Ethanol	107.6±12.4†	376.7±50.3†	9.3±0.2†
Maotai+ DEN	120.2±13.5† ^#^	315.1±53.4† ^#^	8.19±0.1^#^
Ethanol+ DEN	379.6±70†	569.4±105†	13.3±0.1†
DEN	111.3±12.2† ^#^	222.7±24.5† ^#^	9.72±0.1† ^#^
35-week			
Control	50.8±1.8	111.5±9.1	7.9±0.14
Maotai	50.4±7.0	88.9±12.9	7.36±0.2
Ethanol	57.8±10.9	140.7±31.9†	10.8±0.4†
Maotai+ DEN	57.6±10.9^#^	147.2±21.2† ^#^	8.06±0.2^#^
Ethanol+ DEN	146.3±83.4†	474.6±270.9†	15.9±0.5†
DEN	50.8±3.1^#^	150.4±16.0† ^#^	10.1±0.2† ^#^

† Significantly different from Control group, ^#^Significantly different in Maotai+ DEN group and DEN group from ethanol+ DEN group, *P*<0.05.

Malondialdehyde (MDA) is widely used as a lipid peroxidation marker and an oxidative stress parameter, and its levels in serum and tissue reflect the degree of peroxidation damage [21]. The current results showed, at the 3-week, in addition to Maotai group(*P* = 0.647), the MDA levels in other groups were significantly higher than that in control group (*p*<0.001, Table 4), especially in ethanol+ DEN group(*p*<0.01, Table 4). At the 35- week, the Maotai group, Maotai+ DEN group had similary MDA level as that in control group. But the ethanol+ DEN group still showed continuously increasing MDA level (*p*<0.001, Table 4).

### Gene and protein expressional analysis of MT, Nrf2, GCLC and GCLM

**Figure 4:**
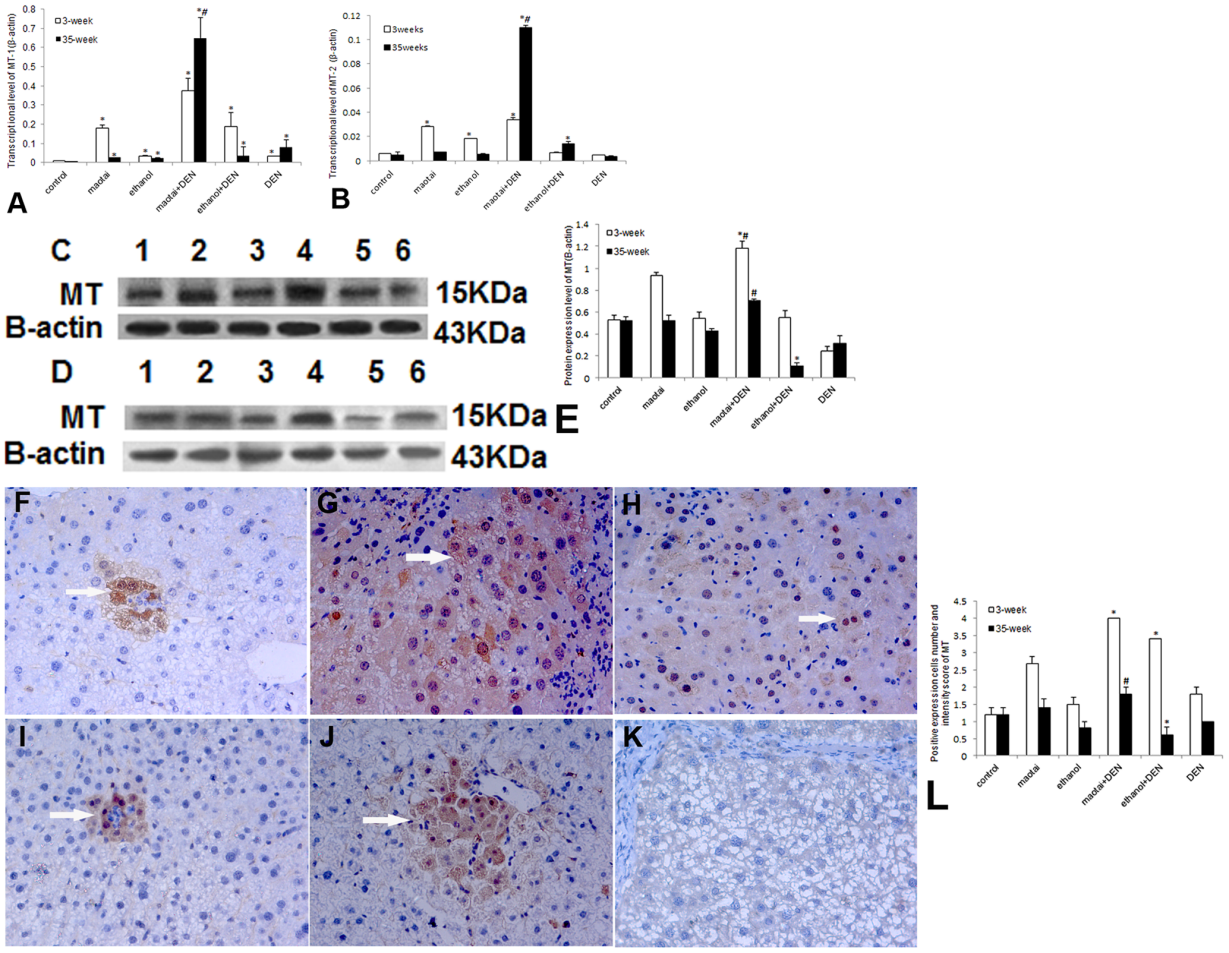
The expression level of MTs in mice liver at 3-week and 35-week. A-B: Real-time RT-PCR analysis of MT-1/2 gene expression. Both the Maotai +DEN group and ethanol+ DEN group produced a significant induction of MT-1 in mRNA level at 3-week. But Maotai +DEN group still produced a dramatic induction of MT-1/MT-2 at 35-week, it was about 19 times of ethanol+ DEN group in MT-1 (*p*<0.01). C–D: Western blot analysis of MTs protein level at 3-week and 35-week. lane 1.Control; lane 2.Maotai; lane 3.Ethanol; lane 4.Maotai+DEN; lane 5.Ethanol+DEN; lane 6.DEN; E: Quantification of MTs level by western blot analysis. The specific up-regulation of MT-1/2 was confirmed through western blotting assay. Maotai+ DEN always had a higher MTs protein level. but it was generally lower in ethanol+ DEN group. F-H, I-K: Light microscopy of MTs expression through Immunohistochemistry in control group, Maotai+ DEN group and ethanol+ DEN group at 3-week and 35-week (400× magnification, allows indicate MTs expression cytolymph and nucleus positive cells); L: Quantification of MTs expression in Immunohistochemistry. Immunohistochemical assay also showed similar MTs protein expression with western blotting assay (*p*<0.01). *Significantly different in Maotai+ DEN group and ethanol+ DEN group from control group, ^#^Significantly different in Maotai+ DEN group from ethanol+ DEN group, *p*<0.05.

**Figure 5:**
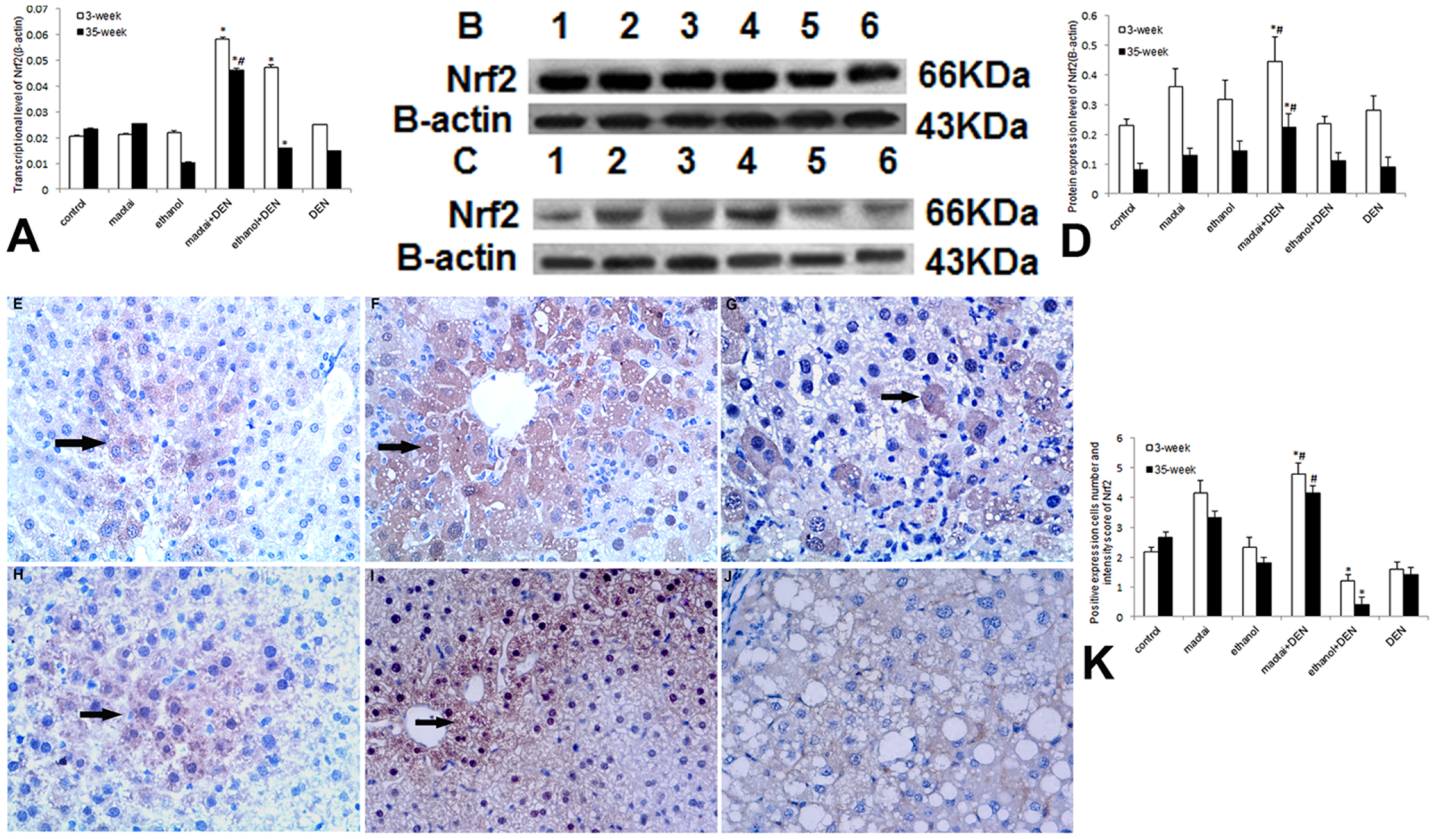
The expression level of Nrf2 in mice liver at 3-week and 35-week. A: Real-time RT-PCR analysis of Nrf2 gene expression. B–C: Western blot analysis of protein level of Nrf2 at 3-week and 35-week. lane 1.Control; lane 2.Maotai; lane 3.Ethanol; lane 4.Maotai+DEN; lane 5.Ethanol+DEN; lane 6.DEN; D: Quantification of Nrf2 level by western blot analysis. E–G, H–J: Light microscopy of Nrf2 expression through Immunohistochemistry in control group, Maotai+ DEN group and ethanol+ DEN group at 3-week and 35-week (400× magnification, allows indicate Nrf2 expression cytolymph and nucleus positive cells); K: Quantification of Nrf2 expression in Immunohistochemistry. Maotai + DEN treatment always enhanced the mRNA and protein expression of Nrf2 at both 3-week and 35-week (*p*<0.05), but ethanol + DEN treatment only transiently increased the mRNA level of Nrf2 at 3-week and the expression level of Nrf2 in this group showed no difference with that in control group at 35-week (*p*>0.05). *Significantly different in Maotai+ DEN group and ethanol+ DEN group from control group, ^#^Significantly different in Maotai+ DEN group from ethanol+ DEN group, *p*<0.05.

**Figure 6:**
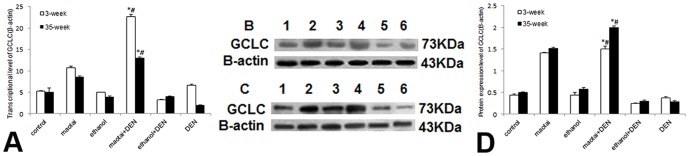
The expression level of GCLC in mice liver at 3-week and 35-week. A: Real-time RT-PCR analysis of GCLC gene expression. B–C: Western blot analysis of protein level of GCLC at 3-week and 35-week. lane 1.Control; lane 2.Maotai; lane 3.Ethanol; lane 4.Maotai+DEN; lane 5.Ethanol+DEN; lane 6.DEN; D: Quantification of GCLC level by western blot analysis. Maotai + DEN group always showed increased GCLC expression level than that in the control group (*p*<0.05), but the ethanol + DEN group always showed lower GCLC expression level than those of control group (*p*<0.05).*Significantly different in Maotai+ DEN group and ethanol+ DEN group from control group, ^#^Significantly different in Maotai+ DEN group from ethanol+ DEN group, *p*<0.05

**Figure 7:**
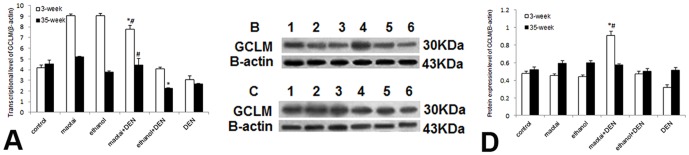
The expression level of GCLM in mice liver at 3-week and 35-week. A: Real-time RT-PCR analysis of GCLM gene expression. B–C: Western blot analysis of protein level of GCLM at 3-week and 35-week. lane 1.Control; lane 2.Maotai; lane 3.Ethanol; lane 4.Maotai+DEN; lane 5.Ethanol+DEN; lane 6.DEN; D: Quantification of GCLM level by western blot analysis. Maotai + DEN could upregulate the mRNA level and protein level of GCLM at 3-week(*p*<0.05), but no any increased expression of GCLM could be seen either in Maotai+ DEN group or ethanol+ DEN group at 35-week (*p*>0.05). *Significantly different in Maotai+ DEN group and ethanol+ DEN group from control group, ^#^Significantly different in Maotai+ DEN group from ethanol+ DEN group, *p*<0.05.

Real-time RT-PCR analysis demonstrated Maotai + DEN group could significantly activate the MT-1 and MT-2 transcription at both 3-week and 35-week (*p*<0.01, Figure 4A and B). and the specific up-regulation of MT-1/2 was confirmed through western blotting assay (*p*<0.01, Figure 4C-E). Ethanol + DEN group showed transient activation in MT-1/2 transcription. The protein levels of MT in this group are generally lower than control group (*p*<0.01, Figure 4 C–E). Immunohistochemical assay also showed similar protein expression of MT (*p*<0.01, Figure 4 F–L). Similarly, Maotai + DEN treatment slightly enhanced the mRNA and protein levels of Nrf2 in both time points (*p*<0.05, Figure 5 A–K), but ethanol + DEN treatment only transiently increased the transcriptional level of Nrf2 within 3-week treatment. The expression of Nrf2 in this group showed no difference with the control level at 35-week time point (*p*>0.05, Figure 5 A–K). In addition, Maotai + DEN group always showed significantly increased GCLC expression level than control group (*p*<0.05, Figure 6 A–D), but the ethanol + DEN group always showed lower GCLC expression level than control group (*p*<0.05, Figure 6 A–D). Maotai + DEN could upregulate the transcriptional level and protein level of GCLM at 3- week (*p*<0.05, Figure 7A–D), but no any increased expression of GCLM could be seen either in Maotai+ DEN group or ethanol+ DEN group at 35-week (*p*>0.05, Figure 7A–D).

## Discussion

Alcoholic beverage consumption has long been recognized as a risk factor for multiple liver diseases, such as fatty degeneration, hepatitis, hepatic fibrosis, cirrhosis, and even HCC [3]–[7]. Epidemiological data also showed that alcohol abuse is associated with the increase of cancer [41], [42]. Present study addressed the different effects of the same dose of Maotai and ethanol on the development and progression of HCC in male C57BL/6J mice initiated by DEN. This study found that ethanol + DEN treatment significantly affected the growth of mice. These mice showed a markedly increase in liver weights. However, Maotai+ DEN treated mice maintained the normal growth, no liver burden could be seen. Serum ALT and AST levels indicated, long-term consumption of Maotai 5 ml/kg/d dose or ethanol didn't cause significant dysfunction of liver. DEN was given twice by intraperitoneal injection also couldn't cause obvious liver damage, may be due to the DNA-repair mechanism [43], [44]. However, ethanol+ DEN treatment could make significantly higher serum ALT and AST levels than in all other groups. The results indicated that ethanol can synergize with DEN and cause serious injury of liver function. So we thought long and moderate drinking Maotai could prevent liver damage caused by ethanol and DEN. The induction of lipid peroxidation by alcohol and its metabolites is also an important pathogenic factor in alcoholic hepatic injury [19]. Therefore, the current study also demonstrated that Maotai+ DEN treatment didn't increase liver MDA content as done with ethanol+ DEN.

Histopathological results showed that ethanol + DEN treatment can aggravate hepatocytes lesions in progressive liver fibrosis, cirrhosis, hepatocytes atypical hyperplasia and the formation of abnormal fission, even changes in the structure of the liver, and eventually leaded to hepatocarcinogenesis.GPC3 is an useful early diagnostic markers for HCC, it's immunohistochemical results also suggested the formation of HCC. Maotai + DEN treatment only cause slight to moderate liver fibrosis, no significant liver damage and no occurrence of HCC could be seen. Our study showed the markedly higher liver damage, liver weight and tumor burden in the ethanol+ DEN treated mice, demonstrated a synergy of ethanol and DEN on hepatocarcinogenesis. But the equal amount of Moutai feed wouldn't form a similar action.

The current study have further demonstrated the possible mechanisms that Maotai could ameliorate the formation of HCC in DEN initiated-mice. Prior studies showed excessive free radical formation and the loss of MTs facilitates hepatocarcinogenesis [19], [23], [45], [46], so we thought that Maotai 's protective mechanism on liver was possibly related with the activation of certain specific anti-oxidation factors or enzymes. In this study, we found both Maotai +DEN treatment and ethanol+ DEN treatment produced a dramatic induction of MT-1 in mRNA level at 3-week. But at 35-week, only Maotai +DEN still produced a dramatic induction of MT-1/MT-2 in mRNA level (Maotai+ DEN elevated the expression of MT-1 97-fold compared with a 5-fold increase in ethanol+ DEN group), even though equal amounts of ethanol were given. Thus, this effect cannot be explained by alcohol content +DEN alone, suggesting that nonalcoholic components in Maotai could be responsible for the beneficial effect. A dramatic induction of MTs with a dose of 5 ml/kg Maotai could be an important adaptive mechanism to reduce liver injury as a result of ethanol+ DEN. It has been reported that MTs initially undergoes transcriptional repression in primary tumors that could be epigenetically silenced at later stages of tumor development, compared with non-cancerous liver tissues[23].But we didn't get a full consistent conclusion with them. We found there still has a certain degree of activated MTs mRNA level in liver carcinoma tissues. Further testing found, ethanol + DEN treatment couldn't upregulate MTs protein level. Maotai+ DEN treatment could always significantly upregulate the mRNA and protein level of MTs, thereby couldbetter protect liver from hepatocarcinogenesis in mice initiated by DEN.

The present study also examined Nrf2 and Nrf2-related gene GCLC and GCLM expression.Nrf2 is a basic leucine zipper transcription factor and plays important roles in mediating cellular antioxidant mechanisms that rescue the liver from a wide variety of toxicants [27], [47]–[49].Accumulating evidence suggests that the Nrf2-ARE pathway exerts diverse biological functions against a variety of liver injury [50], and is proposed to be a potential therapeutic target for liver fibrosis [51]. Nrf2 knockout rats are quite sentitive to liver injury induced by CCl4, DEN and alcohol [52]–[54]. Glutamic acid cysteine ligase (GCL) is a important rate-limiting enzymes, catalyze the synthesis of glutathione, which is a important antioxidant factor. GCLC and GCLM are respectively catalytic subunit and modulatory subunit of GCL, they are regulated by Nrf2 [30], [31]. In this study, we tested the capability of Maotai liquor to induce the Nrf2, GCLC and GCLM for the first time, We want to know whether Nrf2 and its -related gene GCLC and GCLM have implicated for Maotai liquor's beneficial effects. Our study showed, Maotai group and Maotai + DEN group always mildly-moderately increased the expressional level of Nrf2 and GCLC, with the exception of GCLM. However, ethanol+ DEN group, ethanol group and DEN group showed the similary or lower expressional level of Nrf2, GCLC with single compared with control group. Based on these findings, we anticipate that Nrf2 and its-related gene may have implications for Maotai's beneficial effects on the liver. The increased activation of Nrf2 is moderate comparing with MTs, Nrf2 activation might be involved, but it is not a sole mechanism of the protection. The increased MTs could be mainly used to explain the dramatic effects observed in the Maotai+ DEN treated mice. But more work needs to continue to explore the possible mechanism about the beneficial effects of maotai.

In Summary, the present study investigate the effects of Maotai on hepatocarcinogenesis using DEN-initiated mice, compared with isodose ethanol. Our results suggested that Maotai can ameliorate the formation of HCC in DEN-initiated mice, the protection mechanism is possibly related with the activation of anti-oxidation factors, such as MTs, Nrf2 and GCLC.
